# Genomic Analysis of *Penicillium griseofulvum* CF3 Reveals Potential for Plant Growth Promotion and Disease Resistance

**DOI:** 10.3390/jof11020153

**Published:** 2025-02-17

**Authors:** Jianfei Yang, Wenshuai Zang, Jie Chen, Dongying Lu, Ruotong Li, Ciyun Li, Yinhua Chen, Qin Liu, Xiaolei Niu

**Affiliations:** 1 School of Breeding and Multiplication (Sanya Institute of Breeding and Multiplication), School of Tropical Agriculture and Forestry, Hainan University, Sanya 572025, China; yangjianfei1220@163.com (J.Y.); 17861505733@163.com (W.Z.); 20203104280.hainanu@vip.163.com (J.C.); ludongyingi@163.com (D.L.); liruotong1220@163.com (R.L.); lcylcy6400@163.com (C.L.); yhchen@hainanu.edu.cn (Y.C.); 2 National Key Laboratory for Tropical Crop Breeding, Sanya 572025, China; 3 College of Agricultural Engineering, Guangxi Vocational University of Agriculture, Nanning 530007, China

**Keywords:** *Penicillium griseofulvum* CF3, genome sequence, plant growth promotion, disease resistance

## Abstract

*Penicillium griseofulvum* CF3 is a fungus isolated from healthy strawberry soil, with the potential to promote the growth of plants and enhance their resistance to diseases. However, the genome sequence of *P. griseofulvum* CF3 remains unclear. Therefore, we performed the whole-genome CCS sequencing of *P. griseofulvum* CF3 using the PacBio Sequel II platform. The assembled genome comprised 104 contigs, with a total length of 37,564,657 bp, encoding 13,252 protein-coding genes. Comprehensive functional annotation was performed using various BLAST databases, including the non-redundant (Nr) protein sequence database, Gene Ontology (GO), the Kyoto Encyclopedia of Genes and Genomes (KEGG), EuKaryotic Orthologous Groups (KOG), and the Carbohydrate-Active enZymes (CAZy) database, to identify and predict protein-coding genes, tRNAs, and rRNAs. The Antibiotics and Secondary Metabolites Analysis Shell (Antismash) analysis identified 50 biosynthetic gene clusters involved in secondary metabolite production within the *P. griseofulvum* CF3 genome. The whole-genome sequencing of *P. griseofulvum* CF3 helps us to understand its potential mechanisms in promoting plant growth and enhancing disease resistance, paving the way for the application of the CF3 strain in sustainable crop production.

## 1. Introduction

The genus *Penicillium* encompasses over 354 recognized species [1], many of which are known for their production of diverse secondary metabolites (SMs). These metabolites can have significant impacts on both agriculture and medicine, with some being detrimental, while others offer beneficial properties. While certain *Penicillium* species, such as some strains of *P. griseofulvum*, are known postharvest pathogens causing blue mold in stored fruits [2], other isolates exhibit beneficial traits, including plant growth promotion and disease resistance [3].

Fungus-derived bioactive molecules, known as secondary metabolites, play a crucial role in the ecological fitness and environmental adaptation of these organisms. The genes responsible for secondary metabolite biosynthesis are often organized in biosynthetic gene clusters (BGCs) [4]. These metabolites are essential for fungal development, and their production can influence interactions with other organisms, contributing to competition, self-defense, and even symbiotic relationships [5,6]. Filamentous fungi and actinomycetes are particularly prolific producers of antibiotics, with over 40% capable of synthesizing these compounds [5]. However, the specific secondary metabolites and the underlying genetic mechanisms responsible for the beneficial effects of many plant growth-promoting fungi (PGPF), including certain *Penicillium* isolates, are not yet fully understood.

Several *Penicillium* species have demonstrated plant-growth-promoting abilities through various mechanisms. These include enhancing nutrient uptake, producing phytohormones [7,8], and increasing beneficial soil microbes and improving the soil quality [9]. For example, *Penicillium simplicissimum* NL-Z1 enhances legume growth [9], *Penicillium menonorum* KNU-3 boosts cucumber growth [10], and root endophytes like *Penicillium chrysogenum* and *Penicillium brevicompactum* improve plant growth in Antarctic ecosystems by accelerating nitrogen mineralization and enhancing nutrient uptake [7]. *Penicillium oxalicum* CX-1 suppresses the pathogen that causes *Salvia* wilt [11]. In addition to direct growth promotion, some *Penicillium* species can protect plants from pathogens by inducing plant systemic resistance (ISR) [12].

*Penicillium griseofulvum* CF3, a PGPF isolated from healthy strawberry soil, was shown to be a biocontrol agent. It can protect against root diseases in *Aconitum carmichaeli* caused by *Sclerotium rolfsii* and *Fusarium* spp. and promote the growth of this medicinal plant [3]. To elucidate the molecular mechanisms underlying these beneficial properties, we performed the whole-genome sequencing of *P. griseofulvum* CF3. Our research aimed to thoroughly analyze the strain’s genomic architecture, identify crucial genes and biosynthetic gene clusters associated with plant growth promotion and disease resistance, and elucidate fungal metabolic networks, with a particular emphasis on secondary metabolite synthesis pathways. Additionally, we performed comparative genomic analyses with other *Penicillium* strains to gain deeper insights into the mechanistic basis of *P. griseofulvum* CF3’s beneficial traits and evaluate its potential applications as a biocontrol agent in sustainable agriculture.

## 2. Materials and Methods

### 2.1. Strain Culture and DNA Isolation

*P. griseofulvum* CF3, previously isolated from the rhizosphere of healthy strawberry plants, was characterized by the Microbial Resources Laboratory at the College of Natural Resources and Environment, Northwest A&F University [3]. *P. griseofulvum* CF3 was cultured in potato dextrose broth (PDB) medium with shaking at 180 rpm at 28 °C for 7 days. The fungal suspension was filtered using sterile gauze in a laminar flow hood to obtain the mycelium. Fungal DNA was extracted using the cetyltrimethylammonium bromide (CTAB) method [13].

### 2.2. Genome Sequencing and Assembly

Genomic DNA was extracted from *P. griseofulvum* CF3 as described in Section 2.1. Unlike previous studies focusing on the genomic analysis of pathogenic *P. griseofulvum* strains, this study investigated the *P. griseofulvum* CF3 isolate, which has demonstrated significant potential for plant growth promotion and biocontrol [3]. To achieve a high-quality, contiguous genome assembly, we employed the PacBio Sequel II sequencing platform (Pacific Biosciences, Menlo Park, CA, USA), which is known for its long-read sequencing capabilities, enabling a more comprehensive analysis of genomic features compared to short-read sequencing technologies. Whole-genome CCS sequencing was performed using the PacBio Sequel II platform. DNA quality was assessed using three methods: 0.75% agarose gel electrophoresis to assess sample degradation and the fragment size, a NanoDrop spectrophotometer (Thermo Fisher Scientific, Waltham, MA, USA) to determine the DNA purity, and a Qubit system for accurate DNA quantification. After quality control, the experimental steps were performed in accordance with PacBio’s standard protocol, which included sample quality assessment, library construction, library quality control, and sequencing. BluePippin was used to select the required fragment size to generate the sequencing library.

Whole-genome sequencing was used to generate highly accurate circular consensus sequence (CCS) reads. The Hifiasm assembler [14] was used for the de novo assembly of these CCS reads. To further enhance the accuracy of the assembled genome, Pilon [15] was employed for genome polishing using short-read data. The completeness of the final genome assembly was assessed by evaluating the mapping rate of the short-read data using bwa [16] and analyzing the overall genome completeness with a BUSCO analysis. The GenBank accession numbers for both the raw sequencing data and the assembled genome sequence of *P. griseofulvum* CF3 are SRR28599443 and JBLEBL000000000.1, respectively. Furthermore, comparative genomic analyses were performed between *P. griseofulvum* CF3, a reference *P. griseofulvum* strain (PG3, GenBank accession: GCA_001561935.1), and other related *Penicillium* species to provide an evolutionary context and identify unique genomic features of CF3.

### 2.3. Identification of Repeated Sequences and RNAs

We used LTR_FINDER v1.05 [17], MITE-Hunter [18], RepeatScout v1.0.5 [19], and PILER-DF v2.4 [20] to build a repeat sequence library, which was then combined with the Repbase [21] database to form the final library, and we then used the RepeatMasker v4.0.6 [22] software to predict repeat sequences. Transfer RNA (tRNA) genes were predicted with tRNAscan-SE v1.3.1 [23], and ribosome RNA (rRNA) genes were predicted with Infernal v1.1.1 [24]. The GenBlastA v1.0.1 [25] program was used to scan the whole genomes after masking predicted functional genes.

### 2.4. Gene Prediction and Annotation

Gene structure prediction integrates the results from ab initio and homologous protein-based methods. Ab initio prediction was performed using Genscan [26], Augustus v2.4 [27], GlimmerHMM v3.0.4 [28], GeneID v1.4 [29], and SNAP (version 2006-07-28) [30], while homologous protein-based prediction was performed with GeMoMa v1.3.1 [31]. The results were then integrated using EVM v1.1.1 [32]. We utilized Circos for the visualization of the genome data by creating a circular genomic diagram [33].

The predicted proteins were subjected to BLASTP [34] (e-value: 1 × 10^−5^) searches against the NCBI non-redundant protein (Nr) database [35], KEGG [36], KOG [37], Swiss-Prot, and TrEMBL [38]. Blast2GO [39] was used for GO [40] annotation. HMMER [41] was used for Pfam [42] annotation. The pathogenicity was researched by blast against the CAZy [43], PHI [44], and CYPED [45] databases. The secondary metabolism gene cluster was predicted by antiSMASH v.7.1.0 [46].

### 2.5. Genomic Comparison and Phylogenomic Analysis

Synteny analyses between the genomic DNA sequences of *P. citrinum* B9 (GCA_036320845.1), *P. griseofulvum* PG3 (GCA_001561935.1), and *P. griseofulvum* CF3 were performed using MCScanX [47]. The results were visualized using SynVisio [48].

To perform a phylogenetic analysis of *P. griseofulvum* CF3, protein sequences from 13 *Penicillium* species and one outgroup species, *Aspergillus chevalieri* M1, were downloaded from NCBI, including *Penicillium chrysogenum* IBT 35668 (GCA_028827035.1), *Penicillium egyptiacum* (GCA_911456345.1), *Penicillium bovifimosum* IBT 22155 (GCA_028826915.1), *Penicillium digitatum* PHI26 (GCA_000315665.1), *Penicillium italicum* PHI-1 (GCA_000769765.1), *Penicillium brevicompactum* IBT 35665 (GCA_028827555.1), *Penicillium verrucosum* IBT 35672 (GCA_028828655.1), *Penicillium citrinum* B9 (GCA_036320845.1), *Penicillium brasilianum* (GCA_001048715.1), *Penicillium olsonii* (GCA_911174995.1), *Penicillium robsamsonii* IBT 29466 (GCA_028829455.1), *Penicillium canescens* IBT 15451 (GCA_028828765.1), *Aspergillus chevalieri* M1 (GCA_016861735.1), and *P. griseofulvum* PG3 (GCA_001561935.1). Using PHYling [49] with the BUSCO fungal dataset [50], 758 single-copy orthologous genes were identified across 14 fungal genomes for phylogenetic tree construction. The identified orthologous sequences were trimmed using trimAl to remove low-quality regions. The optimal substitution model VT+F+I+R4 was determined using the ModelFinder function in IQ-TREE [51] based on the Bayesian Information Criterion (BIC). Subsequently, a maximum likelihood tree was constructed using 1000 ultrafast bootstrap iterations to assess the support of the branches.

## 3. Results

### 3.1. Genome Sequencing, Assembly, and Genomic Features

**Figure 1:**
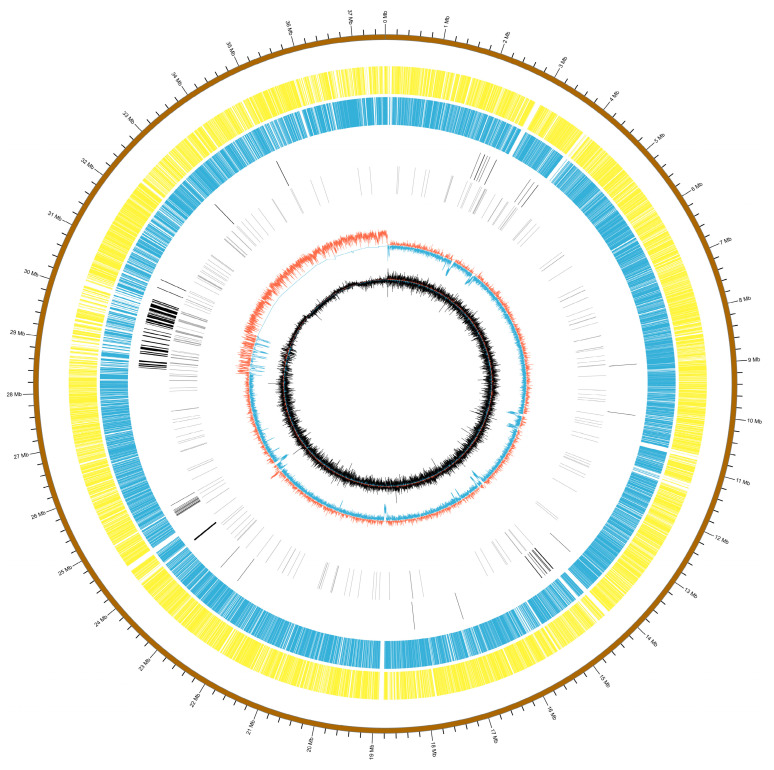
A detailed circular map of the *P. griseofulvum* CF3 genome. From the outermost to the innermost circles, the genome features include forward-strand CDS (yellow), reverse-strand CDS (blue), rRNA (black), tRNA (black), positive GC content (red), negative GC content (blue), positive GC skew (black), and negative GC skew (black; GC shift; GC skew = (G − C)/(G + C)).

Using the PacBio Sequel II long-read sequencing technology, a de novo genome assembly was performed. A total of 56.469 Gb of subreads were generated. These were processed to yield 2.265 Gb of high-quality circular consensus sequencing (CCS) reads. These CCS reads were assembled into contigs using the Hifiasm software (Hifiasm-0.19.8-r603). To further improve the accuracy of the assembly, the resulting contigs were polished using Pilon with short-read data. With a sequencing depth of 150.3×, the final assembled genome had a total length of 37.56 Mb. We mapped the short-read data back to the assembled genome using BWA to assess the completeness and accuracy of the genome assembly. The mapping rate of clean reads to the reference genome was 98.29%. Additionally, genome completeness was assessed using BUSCO v2.0, which identified 286 complete BUSCO genes with a genome completeness score of 98.62%. The final assembled genome of *P. griseofulvum* CF3 had a total length of 37,564,657 bp, GC content of 51.66%, and 13,252 annotated genes. The assembly quality was reflected in its N50 value of 4,472,691 bp. A comparative overview of these genomic features, alongside those of the reference strain, *P. griseofulvum* PG3, is presented in Table 1. This comparison reveals similarities and differences in the genome size, GC content, gene number, and other characteristics between the two strains. Further details of the CF3 genome, including the numbers of tRNA, rRNA, and ncRNA genes, are also provided in Table 1 and Figure 1.

The statistics for repetitive sequences in *P. griseofulvum* CF3 are presented in Table 2. The repetitive sequences in *P. griseofulvum* CF3 account for 4.66% of the whole genome, of which class I repetitive sequences account for 2.98% of the genome, including the Dictyostelium Intermediate Repeat Sequence (DIRS), long interspersed nuclear elements (LINEs), long terminal repeat (LTR) and short interspersed nuclear elements (SINEs), and DNA transposons, which account for 0.62% of the genome. In addition, some repetitive sequences, such as potential host genes, simple sequence repeats (SSRs), and unknown sequences, were predicted.

### 3.2. Gene Functional Annotation

The *P. griseofulvum* CF3 genome contained 13,252 genes, of which 12,092 were annotated in different databases (Table 3). The NR database provided the highest coverage, annotating 12,583 genes (94.95%). The NR database is a non-redundant repository of protein sequences from multiple organisms. TrEMBL annotated 11,901 genes, including all protein sequences that had not yet been manually curated, and it is the translated portion of the EMBL nucleotide sequence database. Pfam annotated 10,600 genes (79.99%); it covers a wide range of protein domains and families. Swiss-Prot annotated 7860 genes (59.31%); it offers higher accuracy compared to TrEMBL. Swiss-Prot is a manually curated and high-quality protein sequence database. In addition, the Gene Ontology (GO) database annotated 9130 genes (68.90%). KOG annotated 6841 genes (51.62%) and KEGG annotated 4029 genes (30.40%) (Table S1).

**Figure 2:**
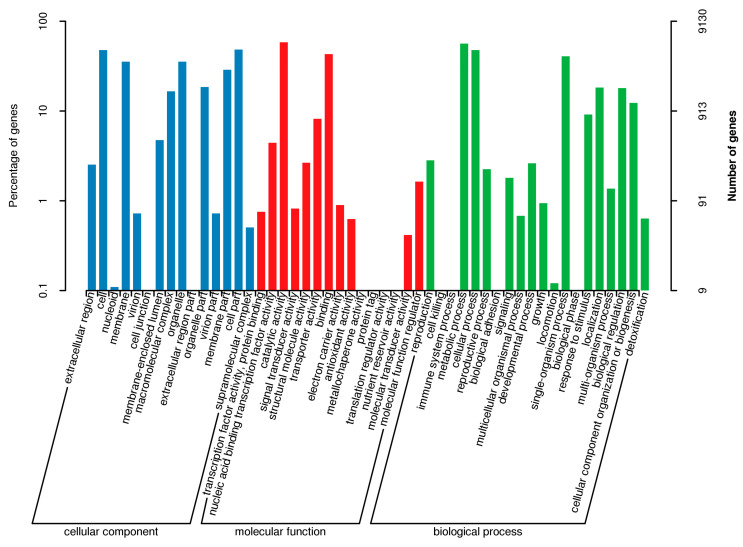
GO annotation of the genome of *P. griseofulvum* CF3.

The GO enrichment analysis showed that 9130 genes (68.90% of the genome) were annotated into three functional categories: biological processes, cellular components, and molecular functions (Table S1). Among the biological processes, 5138 genes were involved in “metabolic processes”, followed by “cellular processes”, “single-organism processes”, “localization”, and “biological regulation”. Among the cellular components, 4411, 4360, 3245, 3233, and 2625 genes were involved in “cell parts”, “cells”, “organelles”, “organelle parts”, and “membrane parts”, respectively. Among the molecular functions, 5342, 3935, 746, 404, and 243 genes participated in “catalytic activity”, “binding”, “transporter activity”, “nucleic acid binding transcription factor activity”, and “structural molecule activity”, respectively (Figure 2).

**Figure 3:**
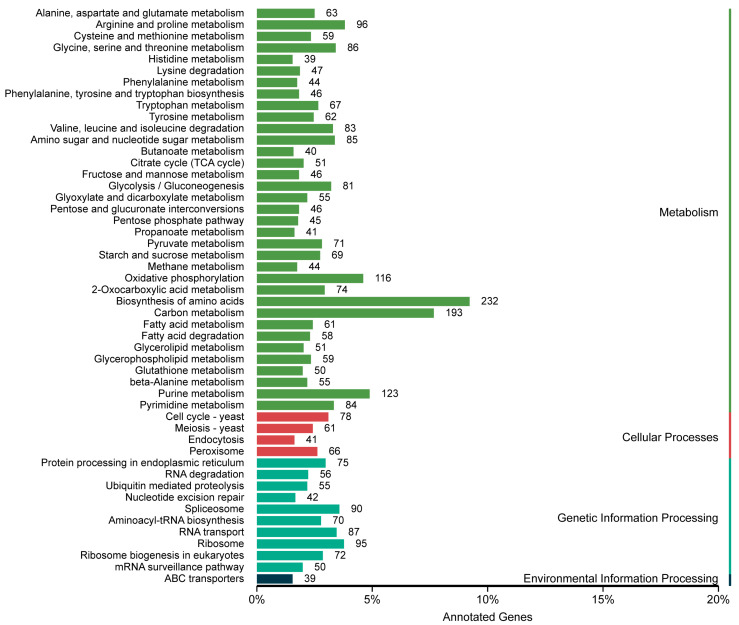
KEGG pathway annotation of the genome of *P. griseofulvum* CF3.

The KEGG enrichment analysis of the *P. griseofulvum* CF3 genome revealed that 4029 genes (30.40% of the genome) were mapped to the KEGG database (Table S1). The top five pathways with the highest numbers of enriched genes were “biosynthesis of amino acids”, “carbon metabolism”, “purine metabolism”, “oxidative phosphorylation”, and “arginine and proline metabolism” (Figure 3).

Studies have shown that VOCs such as sesquiterpenoids (SQTs) influence lateral root growth in the host plants *Populus* and *Arabidopsis* [52]. In addition, many fungi are able to produce sesquiterpenoids with antifungal activity [53,54]. In the CF3 genome, we identified four genes involved in the biosynthetic pathway of sesquiterpenes and triterpenes (*Penicillium*0G005890.1; *Penicillium*0G039390.1; *Penicillium*0G062200.1; *Penicillium*0G132240.1). Sixty-seven genes were enriched for the tryptophan pathway, the main precursor in the synthesis of indole-3-acetic acid (IAA). Previous studies have shown that fungi of the genera *Aspergillus*, *Absidia*, *Mortierella*, *Fusarium*, *Penicillium*, and *Trichoderma* are able to produce IAA for the development of tomato plants [55]. Therefore, CF3 may promote plant growth by synthesizing IAA. Under low nitrogen conditions, the endophytic fungus *Phomopsis liquidambari*, which has a high colonization rate, is able to induce the expression of various genes related to nitrogen uptake and metabolism in rice, thereby increasing the efficiency of nitrogen use [56]. In the CF3 genome, 26 genes were enriched in nitrogen metabolism pathways (Table S1), and the growth promotion of CF3-treated plants may be achieved by increasing the nitrogen use efficiency. Increasing the phosphorus solubility promotes plant growth. Under alkaline conditions (pH ≥ 6.8), organic acids produced by *Trichoderma koningiopsis* NBRI-PR5 dissociate to form phosphorus, which can be utilized by plants, thereby promoting plant growth [57]. Organic acids are involved in a variety of metabolic pathways including glycolysis/gluconeogenesis and the citrate cycle (TCA cycle) (Table S1). Under phosphorus-limited conditions, microorganisms can accumulate polyphosphate as an alternative energy and phosphorus source, which is further converted to orthophosphate by phosphatases such as alkaline phosphatase [58]. KEGG pathways involving phosphatases include thiamine metabolism, riboflavin metabolism, and inositol phosphate metabolism (Table S1). Previous studies have shown that fungi of the genera *Aspergillus*, *Mucor*, *Fusarium*, and *Penicillium* are capable of producing phytase and phosphatase enzymes, which promote plant growth by releasing soluble phosphorus through the solubilization of phosphate [59,60]. *P. griseofulvum* CF3 has the potential to enhance phosphorus solubilization and stimulate plant growth.

**Figure 4:**
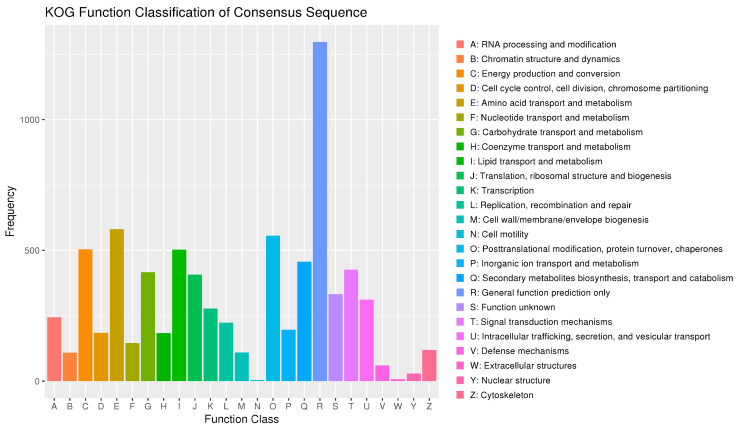
KOG function classification of consensus sequence.

The data included 6841 genes annotated in the KOG database, representing 51.62% of the total gene count (Table S1). The largest KOG category was “general functional prediction only”, with 1298 genes, followed by “amino acid transport and metabolism” (581), “posttranslational modification, protein turnover, chaperones” (556), “energy production and conversion” (504), and “lipid transport and metabolism” (502) (Figure 4).

### 3.3. Carbohydrate-Active Enzymes

**Figure 5:**
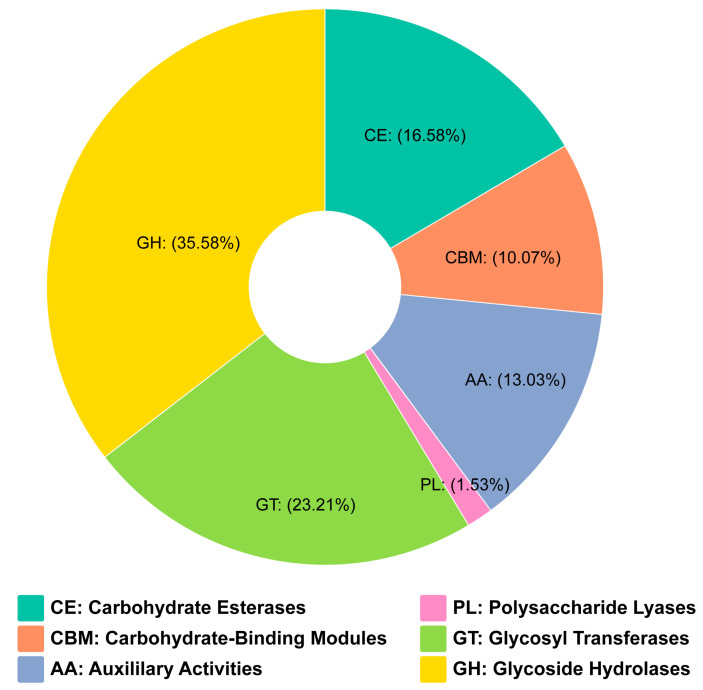
Distribution of carbohydrate-active enzyme (CAZyme) families.

Carbohydrate-active enzymes (CAZymes) are enzymes that synthesize, modify, and break down complex carbohydrates and glycoconjugates, playing crucial roles in various biological processes [43]. To explore the presence of CAZymes in the *P. griseofulvum* CF3 genome, it was aligned with the CAZy database. A total of 698 genes were categorized into six distinct CAZyme families (Table S2). The most abundant enzymatic family identified was glycoside hydrolases, which accounted for 279 CAZyme-encoding genes. The second most prevalent family was glycosyltransferases (GT family), with 182 genes. Additionally, 102 genes were found to encode auxiliary activity (AA), while 79 genes were associated with carbohydrate-binding modules (CBMs). Furthermore, 130 genes encoding carbohydrate esterases (CEs) and 12 polysaccharide lyases (PLs) were identified in the genome (Figure 5).

### 3.4. Pathogen–Host Interaction (PHI) Annotations

A total of 4338 genes (32.73% of the total) were annotated in the PHI database as pathogenicity-related genes (Table S2). Among these, some of these genes result in the reduced virulence of the pathogen. *Penicillium* 0G104030.1 exhibited 99.57% sequence similarity to the PidR gene of *Burkholderia glumae*, the pathogen responsible for rice and onion panicle blight [61]. *Penicillium* 0G009830.1 showed 95.76% similarity to the Moatg8 gene of *Magnaporthe oryzae*, which causes rice blast [62]. *Penicillium* 0G017320.1 exhibited 94.48% similarity to the Hog1 gene of *Cochliobolus heterostrophus*, responsible for southern corn leaf blight [63]. *Penicillium* 0G101300.1 demonstrated 93.3% similarity to the rpoS gene of *Erwinia amylovora*, which causes fire blight in *Eriobotrya japonica* [64]. Additionally, *Penicillium* 0G108380.1 exhibited 93.79% similarity to the GyrA gene of *Burkholderia glumae*, which causes bacterial grain rot in rice; the deletion of this gene led to the increased sensitivity of the pathogen to oxolinic acid [65].

### 3.5. Cytochrome P450 Monooxygenases

Cytochrome P450 monooxygenases (CYPs) are a widely distributed and important enzyme family in biological systems, playing key roles in metabolism, environmental adaptation, and the mechanism of the disease. Conducting a CYP prediction analysis on the *P. griseofulvum* CF3 genome can help us to better understand its metabolic pathways, environmental adaptability, and disease mechanisms. Through a comparison with the CYP database, we identified 578 genes encoding CYPs in the *P. griseofulvum* CF3 genome, accounting for 4.4% of the total genome. The top five CYP families by gene count were CYP51 (98), CYP53 (97), CYP125 (77), CYP504 (51), and CYP149 (36) (Table S2).

### 3.6. Secondary Metabolite Cluster Analysis of P. griseofulvum CF3

The genome-wide analysis of *P. griseofulvum* CF3 using antiSMASH v.7.1.0 identified 50 putative secondary metabolite biosynthetic gene clusters (BGCs) (Table S3). These clusters represent a diverse array of biosynthetic pathways, with type 1 polyketide synthase (T1PKS) clusters being the most abundant (23 clusters), followed by non-ribosomal peptide synthetase (NRPS) clusters (20 clusters). Other identified BGCs included those associated with indoles, fungal arginine-containing cyclic dipeptides (RCDPs), beta-lactone containing protease inhibitors (betalactone), terpene biosynthesis, non-ribosomal peptide metallophores (NRP metallophores), phosphonates, trans-AT PKSs, homoserine lactones (hserlactones), clusters of unknown function (“other”), and heterocystic glycolipid synthase-like PKSs (hglE-KS).

**Figure 6:**
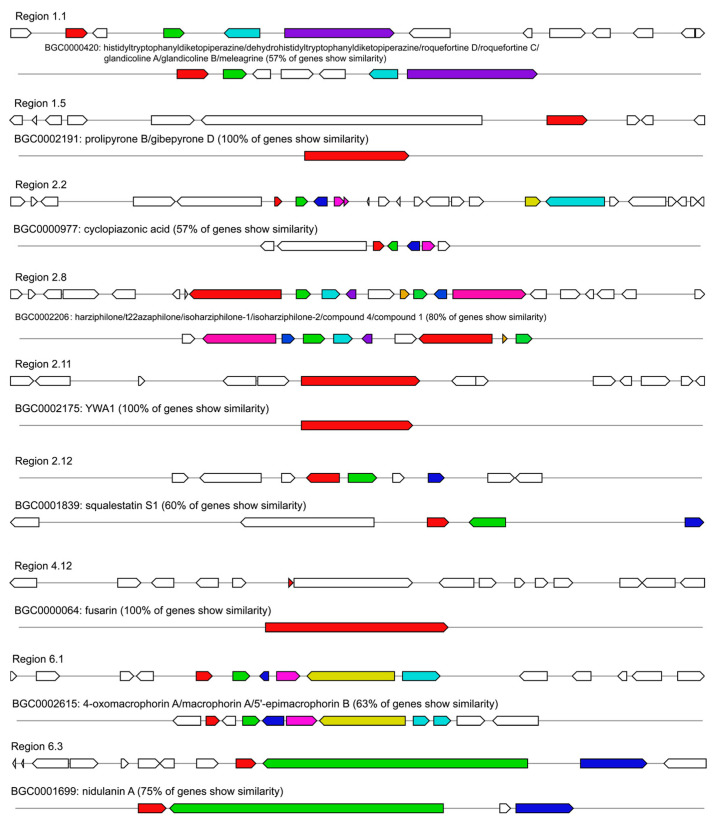
Comparative analysis of biosynthetic gene clusters (BGCs) in *P. griseofulvum* CF3 with known BGCs from the MiBIG database, accessed via the antiSMASH platform. Each region (1.1 to 6.3) represents a putative BGC identified in the *P. griseofulvum* CF3 genome. Arrows represent individual genes, with their orientation indicating the direction of transcription. Genes sharing the same color are homologous and show similarity to genes within a corresponding BGC entry in the MiBIG database. White genes have no significant similarity to any genes in the MiBIG database. The BGC ID and the name of a known compound are listed below each region, along with the percentage of genes within that region showing similarity to the corresponding MiBIG BGC. The comparative analysis was performed using antiSMASH v. 7.1.0, utilizing the MiBIG database for known BGC comparisons.

Twenty-nine of the identified BGCs exhibited varying degrees of similarity to known gene clusters in the antiSMASH database. We focused on BGCs with high similarity to characterized clusters with demonstrated or predicted roles in microbial interactions, plant interactions, or fungal self-protection. Nine BGCs showed more than 50% similarity to known clusters, and three reached 100% similarity based on the overall gene content and domain architecture (Figure 6).

Region 1.5, classified as an “NRPS”, showed 100% identity across the entire cluster to the gene cluster involved in the synthesis of prolipyrone B/gibepyrone D (BGC0002191). Prolipyrone B and gibepyrone D are key compounds in the secondary metabolism of *Aspergillus*, and their function may be to oxidize toxic precursors (gibepyrone A) into less harmful derivatives, thereby assisting in fungal self-protection and regulation [66]. The perfect similarity between the CF3 cluster and BGC0002191 suggests that CF3 possesses the genetic capability to produce prolipyrone B/gibepyrone D or related analogs, which may contribute to its ability to compete with other microorganisms in the soil.

Region 2.11, classified as a “T1PKS”, exhibited 100% similarity to the “YWA1 biosynthetic gene cluster of *Aspergillus oryzae* RIB40” (BGC0002175). This similarity covered the entire cluster and included all enzymes required for biosynthesis. YWA1 is the first intermediate in the biosynthesis of 1,8-dihydroxynaphthalene (DHN) melanin, a pigment that protects fungal cells from host immune responses, oxidative stress, and UV light [67]. Thus, the presence of this conserved BGC in CF3 may contribute to its stress tolerance and survival in the soil environment or help to resist host defense.

Region 4.12, classified as a “T1PKS,” was 100% identical to the “fusarin C biosynthetic gene cluster from *Fusarium verticillioides*” (BGC0000064). The 100% similarity covered the entire cluster, including all key enzymes. Fusarium verticillioides’s fusarin C has significant biological activity and potential ecological functions, playing an important role in the growth of fungi [68]. Given the strong similarity between CF3’s T1PKS and fusarium’s BGCs, CF3 may have the potential to synthesize fusarin-like compounds, which may contribute to its role as a biocontrol agent by inhibiting soilborne pathogens.

In region 1.1, the gene cluster associated with NRPSs and indoles, shares 57% similarity with the gene cluster responsible for the synthesis of histidyltryptophanyldiketopiperazine/dehydrohistidyltryptophanyldiketopiperazine/roquefortine D/roquefortine C/glandicoline A/glandicoline B/meleagrine (BGC0000420). Previous studies have shown that roquefortine inhibits the growth of Gram-positive bacteria [69]. Meleagrin is a downstream product of roquefortine C, which is considered to be a precursor of the compound neoxaline, with antimicrobial activity [70]. Region 2.2, classified as an NRPS, T1PKS, and indole, shares 57% similarity with the cyclopiazonic acid (CPA) biosynthetic cluster (BGC0000977). CPA, produced by *Aspergillus flavus*, is a neurotoxic secondary metabolite and a key virulence factor in the saprophytic lifestyle of this fungus [71]. Region 2.8 is classified as a T1PKS and shares 80% similarity with the synthesizing harziphilone/t22azaphilone/isoharziphilone-1/isoharziphilone-2/compound 4/compound 1 cluster (BGC0002206). Trigazaphilones have high free radical scavenging activity and help to maintain redox homeostasis [72]. Region 2.12, classified as a terpene, shares 60% similarity with the squalestatin S1 biosynthetic cluster (BGC0001839). A number of different squalestatins have been isolated from more than a dozen filamentous fungal taxa, among which SQS1 has antifungal properties. Region 6.1, classified as a T1PKS, shares 63% similarity with the cluster synthesizing 4-oxomacrophorin A/macrophorin A/5”-epimacrophorin B (BGC0002615). Finally, region 6.3, classified as an NRPS, shares 75% similarity with the nidulanin biosynthesis cluster (BGC0001699).

### 3.7. Whole-Genome Synteny Comparisons Between P. griseofulvum CF3 and Other Penicillium Strains

**Figure 7:**
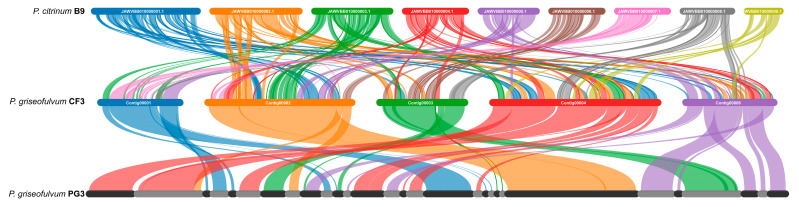
Genome synteny analysis between *P*. *citrinum* CF3 and other *Penicillium* strains.

To elucidate the genomic relationships between *P. griseofulvum* CF3 and other *Penicillium* strains, we performed whole-genome synteny comparisons using *P. citrinum* B9 and *P. griseofulvum* PG3 as references (Figure 7). The comparison between *P. citrinum* B9 and *P. griseofulvum* CF3 revealed synteny of 49.93%. This is visually represented by the relatively sparse and thin connecting bands between the contigs of these two species, indicating a moderate degree of genomic similarity. In contrast, the synteny analysis between *P. griseofulvum* CF3 and *P. griseofulvum* PG3 showed a markedly higher degree of conservation, with 94.90% of their genomes being syntenic. This high percentage is reflected by the numerous and thick connecting bands, signifying substantial conservation in gene content and order between these two strains. These results demonstrate a significant difference in genomic similarity between *P. griseofulvum* CF3 and the two compared strains. The substantially higher synteny with *P. griseofulvum* PG3 compared to *P. citrinum* B9 underscores the closer evolutionary relationship between the two *P. griseofulvum* strains.

### 3.8. Phylogenetic Analyses

**Figure 8:**
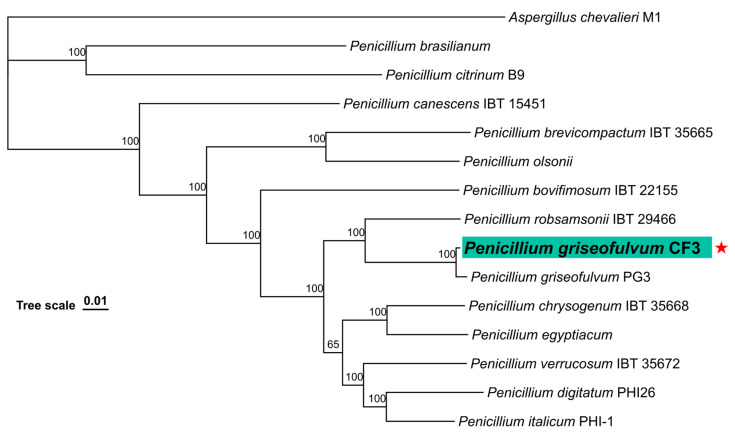
Maximum likelihood phylogenomic tree of *P. griseofulvum* CF3 and related Penicillium species, based on 758 single-copy orthologous genes. *P. griseofulvum* CF3 is highlighted with a star. *Aspergillus chevalieri* M1 was used as the outgroup. Numbers at the nodes represent ultrafast bootstrap support values (1000 replicates). The scale bar indicates the number of substitutions per site.

To determine the phylogenetic relationship of *P. griseofulvum* CF3 within the *Penicillium* genus, a maximum likelihood phylogenetic tree was constructed based on 758 single-copy orthologous genes shared among 13 *Penicillium* species and one outgroup species, *Aspergillus chevalieri* M1 (Figure 8). The phylogenetic analysis strongly supported the placement of *P. griseofulvum* CF3 within a clade that also includes *P. griseofulvum* PG3, with a bootstrap support value of 100%. This close relationship is consistent with their shared species designation. The CF3 and PG3 clade is sister to a clade containing *P. robsamsonii* IBT 29466, *P. bovifimosum* IBT 22155, *P. olsonii*, and *P. brevicompactum* IBT 35665, also with high bootstrap support (100%). The more distant clades include other well-supported *Penicillium* species, such as *P. chrysogenum*, *P. egyptiacum*, *P. verrucosum*, *P. digitatum*, and *P. italicum*. The tree topology, rooted with *A. chevalieri* M1, reflects the established evolutionary relationships within the *Penicillium* genus.

## 4. Discussion

*P. griseofulvum* is a species known for its dual nature, encompassing both postharvest pathogens of fruits [2] and strains with beneficial plant-growth-promoting and biocontrol properties [3]. This study focused on *P. griseofulvum* CF3, a strain isolated from healthy strawberry soil and previously demonstrated to suppress root diseases and enhance the growth of *Aconitum carmichaelii* [3]. Our objective was to decipher the genomic features of CF3 that contribute to these beneficial traits, potentially paving the way for its application in sustainable agriculture. The results reveal the complex interplay among nutrient mobilization strategies, phytohormone production potential, and a remarkably diverse arsenal of secondary metabolite biosynthetic gene clusters (BGCs).

Phylogenetic analysis, using single-copy orthologous genes, confirmed CF3’s classification within the *P. griseofulvum* clade, closely related to the reference strain PG3 (Figure 7). This is consistent with their shared morphological and physiological characteristics. However, despite the high overall genome synteny (94.90%), comparative genomics revealed subtle but potentially crucial differences. While the overall genome sizes are similar (CF3: 37.56 Mb, PG3: 29.14 Mb), CF3 exhibits a slightly higher gene count (13,252 vs. 9629) and variations in the numbers of tRNA, rRNA, and ncRNA genes (Table 1). These differences, concentrated within the 5.1% non-syntenic regions, likely reflect adaptation to distinct ecological niches and may underpin the observed functional differences between the pathogenic PG3 [2] and the beneficial CF3 [3].

The KEGG pathway enrichment analysis provided insights into the potential mechanisms by which CF3 promotes plant growth. A key finding was the significant enrichment of genes involved in the “biosynthesis of amino acids” (193 genes; Figure 3), a sub-branch of nitrogen metabolism. This suggests that CF3 may enhance the nitrogen utilization efficiency in the host plant, a trait common to many plant-growth-promoting fungi (PGPF) [12]. This could involve mechanisms such as improved nitrogen uptake, assimilation, or even nitrogen fixation, although further research is needed to confirm the latter. The role of *Penicillium* and *Phomopsis* species in enhancing nitrogen availability has been demonstrated in other studies [7,56], providing further support for this hypothesis.

In addition to nitrogen metabolism, the enrichment of 67 genes in the tryptophan metabolic pathway (Figure 3) points to a potential role for indole-3-acetic acid (IAA) production. IAA is a key phytohormone that regulates various aspects of plant growth and development [73]. Many *Penicillium* species, along with other fungal genera like *Aspergillus*, are known to produce IAA and promote plant growth through this mechanism [68,74,75], making it highly likely that CF3 utilizes a similar strategy.

Phosphorus availability is often a limiting factor for plant growth. Our analysis revealed multiple lines of genomic evidence suggesting that CF3 can enhance phosphorus solubilization. Pathways related to glycolysis/gluconeogenesis, the citrate cycle (TCA cycle), and thiamine, riboflavin, and inositol phosphate metabolism were all enriched (Figure 3). These pathways contribute to the production of organic acids and phosphatases, enzymes known to solubilize inorganic phosphate, making it more accessible to plants [8,57,59,60]. This is a well-established mechanism of plant growth promotion by fungi [12].

The most striking aspect of CF3’s genome was the presence of 50 BGCs identified by antiSMASH (Table S3), indicating an exceptional capacity for secondary metabolite production. This includes 23 T1PKS and 20 NRPS clusters, suggesting the potential for a wide array of bioactive compounds with antifungal, antibacterial, and other activity. The identification of BGC regions with 100% similarity to known clusters responsible for the synthesis of prolipyrone B/gibepyrone D (region 1.5), YWA1 (region 2.11), and fusarin (region 4.12) is particularly noteworthy. Prolipyrone B and gibepyrone D exhibit antifungal activity [66], and YWA1 is involved in the synthesis of DHN-melanin, a pigment that protects fungal cells from stress, including host defenses [67]. The presence of the fusarin cluster, while often associated with mycotoxin production [68], highlights the complex ecological roles of these compounds, which may also contribute to niche competition.

Furthermore, several other BGC regions showed significant similarity (>50%) to clusters involved in the production of compounds with known antimicrobial activity. These include roquefortine/meleagrin (region 1.1, 57% similarity; antibacterial) [70], cyclopiazonic acid (region 2.2, 57% similarity; potentially involved in saprophytic fitness/virulence) [71], trigazaphilones (region 2.8, 80% similarity; antioxidant) [72], and squalestatin S1 (region 2.12, 60% similarity; antifungal). This remarkable diversity of potentially bioactive compounds strongly suggests that CF3 employs a multi-pronged strategy for pathogen suppression and competition in the rhizosphere. The pathways related to sesquiterpene and triterpene were enriched. The volatile sesquiterpenoids produced by fungi play a role in inducing lateral root development in plants and exhibit antifungal activity [52].

The high number of CAZyme-encoding genes (698; Table S2), especially GHs and GTs, suggests that CF3 is proficient in degrading complex carbohydrates. This could support a saprophytic lifestyle, but it also likely plays a significant role in nutrient cycling and competition with other microorganisms in the rhizosphere [43]. While the potential for plant cell wall degradation cannot be entirely ruled out, the overall genomic and phenotypic evidence points towards a beneficial, rather than pathogenic, interaction with plants.

## 5. Conclusions

The genome of *P. griseofulvum* CF3 reveals the remarkable convergence of mechanisms that contribute to its plant-beneficial traits. The potential for enhanced nutrient mobilization, phytohormone production, and the synthesis of a diverse array of secondary metabolites positions CF3 as a highly promising candidate for development as a biofertilizer and biocontrol agent. This study provides critical insights into the genetic basis of these beneficial properties, laying the groundwork for future research on and the application of CF3 in sustainable agricultural practices, reducing the reliance on chemical inputs and promoting environmentally friendly crop production.

## Figures and Tables

**Table 1:** Genome statistics of *P. griseofulvum* strains CF3 and PG3.

Characteristic	*P. griseofulvum* CF3	*P. griseofulvum* PG3
Genome size (bp)	37,564,657	29,140,916
N50 (bp)	4,472,691	2,267,136
GC content (%)	51.66	47.51
Gene number	13,252	9630
Average gene length (bp)	1807.62	1733.25
tRNA	401	316
rRNA	262	256
ncRNA	127	112

**Table 2:** Statistical results of repeat sequences in the *P. griseofulvum* CF3 genome.

Type	Number	Length (bp)	Percentage (%)
ClassI	528	1,119,068	2.98
ClassI/DIRS	1	43	0.00
ClassI/LINE	26	9629	0.03
ClassI/LTR	38	57,642	0.15
ClassI/LTR/Copia	18	3207	0.01
ClassI/LTR/Gypsy	264	385,986	1.03
ClassI/PLE|LARD	175	670,579	1.79
ClassI/SINE	4	548	0.00
ClassI/Unknown	2	1665	0.00
ClassII	253	232,223	0.62
ClassII/Helitron	12	33,493	0.09
ClassII/MITE	30	6388	0.02
ClassII/Maverick	1	58	0.00
ClassII/TIR	201	191,872	0.51
ClassII/Unknown	9	627	0.00
PotentialHostGene	142	253,793	0.68
SSR	11	6975	0.02
Unknown	747	158,602	0.42
Total	934	1,749,114	4.66

**Table 3:** Annotation statistics for *P. griseofulvum* CF3 genome across various databases.

Database	Number	100 ≤ Length < 300	Length ≥ 300
GO	9130	1841	7128
KEGG	4029	791	3162
KOG	6841	1180	5591
Pfam	10,600	2107	8385
Swiss-Prot	7860	1363	6397
TrEMBL	11,901	2841	8822
NR	12,583	2974	9369
Annotated in all databases	12,902	3073	9588

## Data Availability

The datasets presented in the study can be found online: https://www.ncbi.nlm.nih.gov/bioproject/PRJNA1198730/ (accessed on 15 October 2024). GenBank Accession Number: JBLEBL000000000.1. All other data are provided in the article’s Results section and Supplementary Files.

## Supplementary Materials

The following supporting information can be downloaded at: https://www.mdpi.com/article/10.3390/jof11020153/s1, Table S1: General database annotation of *Penicillium griseofulvum* CF3 genome; Table S2: Special database annotation of *Penicillium griseofulvum* CF3 genome; Table S3: Prediction of secondary metabolite gene clusters in *Penicillium griseofulvum* CF3 genome.
